# Investigating the Impact of Zinc Oxide Nanoparticles and Folic Acid on Neuronal Markers in a Rat Model of Nanotoxicity

**DOI:** 10.1155/jt/1695369

**Published:** 2025-09-23

**Authors:** Shreen Deeb Nusair, Rand Ghaleb Almbaidin, Nour Ahmad Al-Sawalha

**Affiliations:** ^1^Department of Clinical Pharmacy, Faculty of Pharmacy, Jordan University of Science and Technology, P.O. Box 3030, Irbid 22110, Jordan; ^2^Department of Forensic Medicine, Toxicology and Forensic Sciences, Faculty of Medicine, Jordan University of Science and Technology, Irbid, Jordan

**Keywords:** glial fibrillary acid protein, monoamine oxidase A, myelin basic protein, neurofilament light chain, neuronal biomarkers

## Abstract

Exposure to zinc oxide nanoparticles (ZnONPs) is more likely due to their wide utilization in the food and pharmaceutical sectors. Therefore, serum neuromarkers and hippocampal tissue were examined for the potential prophylactic impact of folic acid in four groups of rats, each consisting of 10 animals. The first group had 150 mg/kg ZnONPs orally every day for 2 weeks. The second group received 10 mg/kg of folic acid intraperitoneal (ip) for 1 week, followed by ZnONPs daily for 2 weeks. The third group received folic acid only, while the control group was given distilled water. At the end of the experiments, hippocampi were examined, and serum concentrations were measured for glial fibrillary acidic protein (GFAP), myelin basic protein (MBP), monoamine oxidase A (MAOA), and neurofilament light polypeptide (NEFL). ZnONPs-exposed animals exhibited significantly lower levels of GFAP and MBP (*p* < 0.05 and *p* < 0.001, respectively) compared to all groups, while the same overdosed animals showed significantly higher levels of MAOA compared to the group that received folic acid prophylaxis (*p* < 0.001). NEFL levels did not significantly differ among all groups. Histopathological analysis revealed neurodegeneration in the ZnONPs-exposed group, characterized by neuronal shrinkage, hyperchromatic nuclei, vacuolated cytoplasm, and cell loss. Folic acid partially mitigated these effects, preserving Nissl granules and reducing pyknotic changes, though some ghost cells persisted. In summary, the positive impact of folic acid on reducing ZnONPs toxicity is promising to be further investigated as a preventive measure against nanoparticle-induced neurotoxicity.

## 1. Introduction

Zinc oxide nanoparticles (ZnONPs) have a molecular weight of 81.38 g/mol and are known for their white color and absence of odor [1]. Through altering the size (typically within the range of 10 and 100 nm), shape, or coating of the particles, the characteristics of ZnONPs can be modified. ZnONPs exhibit diverse functions in various fields such as optics, catalysis, electronics, and photochemistry. Additionally, they have demonstrated effectiveness in food preservation, drug delivery, as well as in the elimination of bacteria and cancer cells [1].

Annually, the worldwide production of ZnONPs is approximately ranging from 0.1 to 1.2 million tons [2]. As the use of ZnONPs expands, the potential for human exposure through the primary routes (i.e., inhalation, skin contact, and ingestion) also increases. ZnONPs have the capability to dissolve in external mediums, resulting in the formation of Zn^2+^ ions. These ions can enter cells by passive diffusion across the plasma membrane, which is a critical process in cellular toxicity [3]. In addition to being taken through passive diffusion or membrane channels, ZnONPs can be internalized through receptor-mediated endocytosis, where they are engulfed by the cell [2].

The existing research on the potential toxic effects of ZnONPs has highlighted their cytotoxic effects, including alterations in cell viability, disruptions in cytoskeletal structure, and increased production of reactive oxygen species (ROS), using in vitro cell culture models. However, there are a scarcity of data concerning their toxicity on the genetic material, the nervous system, embryonic development, and other related areas [2].

While many studies have examined the neurotoxic effects of ZnONPs at the molecular and cellular levels, currently, there is no research on the protective role of folic acid in mitigating the neurotoxicity of these nanoparticles. A previous study has demonstrated that supplementation with quercetin can alleviate cerebellar neurotoxicity induced by ZnONPs [4]. Another research study recorded impairments in ability to move and an increase in anxiety levels in adult mice that were exposed orally to ZnONPs. In addition, the exposure to ZnONPs had notable impacts on the transcriptional signaling pathways that are associated with the production of melatonin and various metabolic processes [5].

Folic acid, or called vitamin B9, is an essential B-vitamin that is a critical substance for the development and functioning of the nervous system. It is essential for the synthesis of DNA and RNA, which maintain integrity of cells, including nerve cells [6]. Folic acid participates in the regulation of homocysteine levels, preventing the accumulation of this amino acid which in excessive amounts can be detrimental to nerve cells. Higher levels of homocysteine have been associated with developing neurodegenerative disorders like Alzheimer's disease [6].

Research reported that folic acid can mitigate the neurotoxic effects caused by lead exposure in young rat offspring [7]. Scientists have not yet fully comprehended the specific mechanisms in which folic acid protects against neurotoxicity. Nevertheless, it is hypothesized that its involvement in the synthesis of DNA and RNA, as well as its capacity to regulate levels of homocysteine, may play a part in its protective properties.

The present study examined the potential neurotoxic effect of ZnONPs on adult rats and assessed folic acid protective effect. This was achieved by analyzing four neuronal biomarkers in serum: glial fibrillary acidic protein (GFAP), monoamine oxidase A (MAOA), myelin basic protein (MBP), and neurofilament light polypeptide (NEFL). In addition, hippocampi from rats were examined for histopathological changes. The selected biomarkers as well as neuronal tissue serve as key indicators of the effects of ZnONPs on the nervous system and the potential protective properties of folic acid.

## 2. Materials and Methods

### 2.1. Ethical Use of Animals

The animal experiments followed ethical guidelines outlined in the National Guide for the Animal Care and Use of Laboratory Animals and were approved by the Animal Care and Use Committee (ACUC) at Jordan University of Science and Technology (JUST) with the approval number 18/2/3/16. The national guidelines are in accordance with Guidance on the operation of the Animals (Scientific Procedures) Act 1986 and associated guidelines. All necessary precautions were taken to ensure laboratory health and safety during the experimental procedures described in this study.

### 2.2. Animal Housing and Acclimatization Conditions

Forty adult male Sprague-Dawley rats, 12 weeks and weighing between 248 and 295 g, were divided into four groups of 10 and housed in the Animal Care Unit. An assigned veterinarian monitored the health of the rats throughout the study. The rats were given 1 week to adapt after being transferred to stainless-steel wire cages and a bedding of hardwood chips. They had free access to water and standard chow. The temperature of the experimental room was maintained at approximately 25 ± 2°C and humidity of 26% with a twelve-hour light: dark cycle.

### 2.3. ZnONPs and Folic Acid Treatment Protocols

Four groups of rats were treated as the following: The first group received distilled water for 1 week, followed by a daily oral administration of 150 mg/kg body weight of ZnONPs for 2 weeks. The second group received an intraperitoneal (ip) injection of 10 mg/kg folic acid for 1 week prior to the ZnONPs treatment, which continued for 2 weeks. The third group received folic acid for 1 week and then distilled water for 2 weeks. The control group received only distilled water for the 3-week duration of the study. All doses were freshly prepared in distilled water upon administration to the rats. The doses were adjusted from previous studies on ZnONPs [8, 9] and folic acid [7]. The animals were monitored daily, with a specific focus on observing any clinical or behavioral changes within 1 hour after each dose. No changes were observed or detected.

### 2.4. Processing of Samples

About 2 mL of blood samples were taken retro-orbitally by the end of the experiment [10]. The samples were allowed to coagulate for 30 min at room temperature. Then, samples were centrifuged at 3000 × g for 10 min in refrigerated centrifuge at 4°C and stored in aliquots for later analysis at −20°C [11].

### 2.5. Measuring GFAP, MAOA, MBP, and NEFL Using ELISA

The enzyme-linked immunosorbent assay (ELISA) was conducted to measure the neuro biomarkers GFAP, MAOA, MBP, and NEFL. For this purpose, rat ELISA kit was purchased from MyBioSource, USA (GFAP MBS2505953; MAOA MBS2703950; MBP MBS450557; and NEFL MBS763661). Optimum serum dilution was determined following a pilot run that was conducted for the three kits using 1:1, 1:2, 1:4, and 1:9 sample dilutions. Based on this, the dilution of 1:9 was selected for all assays except NEFL, which required 1:2 dilution. Based on the instructions provided by the manufacturer [12], the dilution buffer and the wash solution were prepared 1:20 in distilled water. Standard concentrations for GFAP were 0, 0.5, 1.0, 2.0, 4.0, and 8.0 pg/mL; standard concentrations for MOAO were 0, 2.5, 5.0, 10.0, 20.0, and 40.0 U/mL; standard concentrations for MBP were 0, 0.156, 0.312, 0.625, 1.25, 2.5, 5.0, and 10.0 ng/mL; and standard concentrations for NEFL were 0, 15.625, 31.25, 62.5, 125, 250, 500, and 1000 pg/mL.

A total of 50 μL per each serum sample was placed in a well of a 96-microwell plate, followed by the addition of 40 μL of diluent per well. Then, 100 μL of each kit specific conjugated antibodies was added to each well and incubated for 60 min at 37°C (GFAP and MAOA), and 90 min for MBP and NEFL. After incubation, the wells were washed three times with washing solution and tapped gently against a paper towel. Each kit substrate solution was then added to each well and incubated in a dark place at 37°C for 15 min for GFAP and MAOA, while MBP required 30 min, and NEFL required 20 min. After incubation, stop solution was added to each well, changing the color from blue to yellow. Samples were processed in triplicates and measured at 450 nm wavelength using the microplate reader Multiskan Go spectrophotometer (model 1510, Thermo Fisher, UK).

### 2.6. Histopathological Examination and Semiquantitative Scoring

The hippocampi from two rats per group were preserved in 10% formalin. The samples were then sectioned into 4 μm slices using an HM 325 rotary microtome (Thermo Scientific, USA). Section processing followed a previously established protocol [13, 14]. Finally, the processed sections were mounted on glass slides and stained with hematoxylin and eosin (H&E). The slides were air-dried and subsequently examined using a B-290TB digital light microscope (Optika, Italy).

Semiquantitative scoring of tissue was adopted with modification from a previously published protocol [15]. Tissue assessment was performed by evaluating 5 nonadjacent H&E-stained sections per animal at 400× magnification and scoring 3 random fields per section, avoiding edges/artifacts. Two independent observers examined the slides. Discrepancies of > 1 point difference were resolved by reexamination. Cell counting was determined using the following equation: % affected cells = (number of abnormal cells)/(total granule cells in field) × 100. Combined Pathology Index was calculated per animal using the following equation: total score = (ghost cells + pyknosis + karyolysis)/3. Please refer to Table 1 for scoring scale and criteria for dentate gyrus granule cells. It is recommended for future research to perform immunohistochemistry (e.g., TUNEL for apoptosis and NeuN for neuron loss).

### 2.7. Statistical Analysis of Data

The data were calculated as mean ± standard deviation (M ± SD) [16]. All data was analyzed using ANOVA in GraphPad Prism Version 8.0.1 (GraphPad Software, USA, 2018) [17]. Tukey's contrast analysis was used to determine significant differences between and within groups [18]. The Shapiro–Wilk test was conducted to check the normality of the data [10, 19]. The value *p* < 0.05 was considered significant with a confidence interval (CI) of 95%.

## 3. Results

### 3.1. Measurements of GFAP, MAOA, MBP, and NEFL

**Figure 1:**
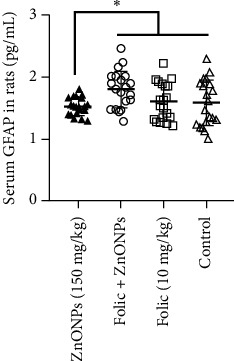
Mean concentrations (pg/mL) of GFAP in serum of 4 groups of rats. From left to right: a group had 150 mg/kg ZnONPs; a group had 10 mg/kg folic acid for 1 week and then 150 mg/kg ZnONPs for 2 weeks; a group had folic acid 1 week and then distilled water for 2 weeks; and the control had distilled water for 3 weeks. Whiskers represent one standard deviation. ^∗^*p* < 0.05.

The average levels of serum GFAP were measured in the four different groups of rats: ZnONPs overdosed rats, rats with folic acid prior to ZnONPs overdose, rats treated with folic acid only, and control rats given distilled water. The concentration of serum GFAP was found 1.53 ± 0.14 pg/mL in ZnONPs overdosed rats, 1.81 ± 0.30 pg/mL in rats with folic acid prophylaxis, 1.61 ± 0.30 pg/mL in rats treated with folic acid only, and 1.58 ± 0.37 pg/mL in control rats. The animals without prophylaxis showed significantly lower levels of serum GFAP compared to the animals with prophylaxis, as well as the animals treated with folic acid only and the control animals (*p* < 0.05). The levels of serum GFAP are depicted in Figure 1 for the challenged rats.

**Figure 2:**
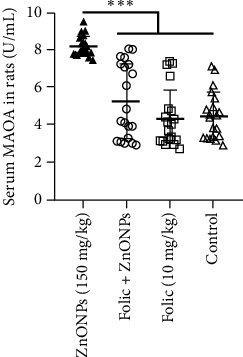
Mean concentrations (U/mL) of MAOA in serum of 4 groups of rats. From left to right: a group had 150 mg/kg ZnONPs; a group had 10 mg/kg folic acid for 1 week and then 150 mg/kg ZnONPs for 2 weeks; a group had folic acid 1 week and then distilled water for 2 weeks; and the control had distilled water for 3 weeks. Whiskers represent one standard deviation. ^∗∗∗^*p* < 0.001.

The mean concentration of serum MAOA was 8.16 ± 0.54 U/mL in the rats exposed to an overdose of ZnONPs, while rats that received folic acid prophylaxis had a lower mean concentration of 5.25 ± 1.99 U/mL. The group that received folic acid treatment alone had a mean concentration of 4.32 ± 1.56 U/mL, while the control had a mean concentration of 4.46 ± 1.26 U/mL. The rats without folic acid prophylaxis had significantly higher levels of serum MAOA compared to all other groups, including the control group (*p* < 0.001).  Figure 2 illustrates the serum levels of MAOA in the four groups.

**Figure 3:**
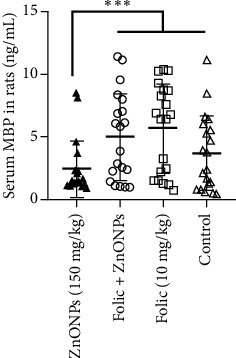
Mean concentrations (ng/mL) of MBP in serum of 4 groups of rats. From left to right: a group had 150 mg/kg ZnONPs; a group had 10 mg/kg folic acid for 1 week and then 150 mg/kg ZnONPs for 2 weeks; a group had folic acid for 1 week and then distilled water for 2 weeks; and the control had distilled water for 3 weeks. Whiskers represent one standard deviation. ^∗∗∗^*p* < 0.001.

The mean concentrations of serum MBP were found 1.62 ± 0.68 ng/mL in rats exposed to ZnONPs overdose, 5.02 ± 3.49 ng/mL in rats with prophylaxis, 5.72 ± 3.53 ng/mL in rats treated with folic acid only, and 3.72 ± 3.02 ng/mL in the control group. The rats exposed to ZnONPs without folic acid prophylaxis had significantly lower levels of serum MBP compared to rats with prophylaxis, rats treated with folic acid only, and the control group (*p* < 0.001). Please refer to Figure 3 for a visual representation of the serum MBP levels in rats.

**Figure 4:**
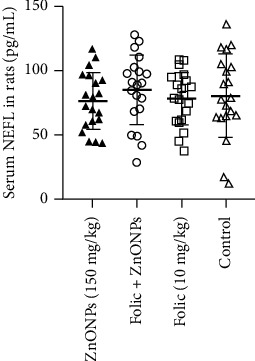
Mean concentrations (pg/mL) of NEFL in serum of 4 groups of rats. From left to right: a group had 150 mg/kg ZnONPs; a group had 10 mg/kg folic acid for 1 week and then 150 mg/kg ZnONPs for 2 weeks; a group had folic acid for 1 week and then distilled water for 2 weeks; and the control had distilled water for 3 weeks. Whiskers represent one standard deviation.

The average concentrations of serum NEFL were measured at 76.50 ± 22.34 pg/mL in ZnONPs intoxicated rats, 84.94 ± 27.13 pg/mL in rats with prophylaxis, 78.20 ± 20.62 pg/mL in rats treated with folic acid only, and 80.60 ± 32.54 pg/mL in the control group. Statistical analysis showed that there were no significant differences in NEFL levels between all groups. For a visual presentation of the NEFL levels in the serum of rats, please refer to Figure 4.

### 3.2. Histopathological Findings

**Figure 5:**
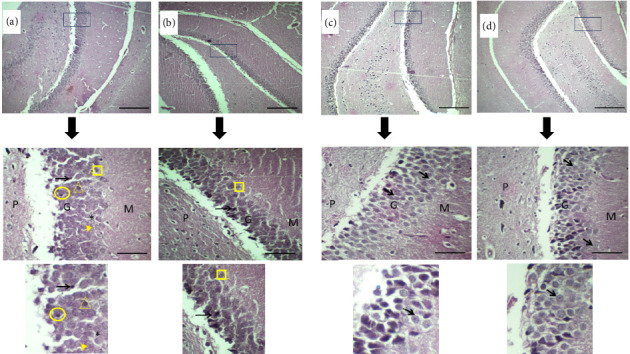
The dentate gyrus in all panels are from rat hippocampus of the challenged 4 groups: (a) ZnONPs group; pleopathologic changes of many of granule cells' nuclei as well as vacuolated cytoplasm (asterisk). There are shrunken neurons, with hyperchromatic nuclei (long arrows) and vacuolated cytoplasm (asterisk). Some granule cells have vesicular nuclei with clogged marginated chromatin and prominent nucleoli (circle). Others have ghost changes (rectangle), while some have karyolysis (triangle). Generally, the granule cells are stained acidophilic relative to that from other groups including control. (b) Folic + ZnONPs group; granule cells show pyknosis (arrow head), well-formed Nissl's granules, and vesicular nuclei. Some cells have ghost changes (rectangle). (c) Folic group and (d) control group; granule cell-layer neurons (short arrow) are evenly arranged and uniform in size. Each neuron has a rounded central vesicular nucleus with prominent nucleolus. The cytoplasm contains prominent basophilic cytoplasmic Nissl's granules and is surrounded by thin neuropil. The dentate gyrus contains 3 layers from left to right, the molecular layer (M), the granule cell layer (G), and the polymorphic layer or called the hilus (P). All identified features (e.g., arrows/circles) are reference established in Bancroft's Theory and Practice of Histological Techniques. Sections were stained by H&E and examined at x400. Scale bars represent 100 μm at 400 magnification and 500 μm at 100× magnification.

The dentate gyrus was examined across the four experimental groups, revealing distinct histopathological alterations, which are illustrated in Figure 5. Neurodegenerative changes were prominent in ZnONPs Group (A), including shrunken neurons with hyperchromatic condensed nuclei and vacuolated cytoplasm (suggesting cellular damage). Granule cells exhibited vesicular nuclei with marginated chromatin and prominent nucleoli (possible early degenerative changes). In addition, ghost cells (necrotic neurons with faint cellular outlines) and karyolysis (nuclear dissolution) were observed. Granule cells showed increased acidophilia, consistent with nucleic acid loss and cytoplasmic protein accumulation from cell injury, suggesting neurotoxicity. Moderate neuroprotection was observed in Folic + ZnONPs Group (B) compared to the ZnONPs group. Some neurons showed pyknosis (irreversible nuclear condensation) with basophilic cytoplasm, but Nissl's granules (indicating intact protein synthesis) were preserved. Vesicular nuclei were present, but a few ghost cells remained, suggesting partial mitigation of damage by folic acid. Normal histoarchitecture with healthy, uniformly arranged granule cells is seen in both folic group (C) and control group (D). Neurons of both groups displayed round, vesicular nuclei with prominent nucleoli and well-defined Nissl's granules. The neuropil (supporting tissue) appeared intact, with no signs of degeneration. Generally, the dentate gyrus layers (molecular, granule cell, and polymorphic/hilus) were well-organized, confirming structural integrity.

**Figure 6:**
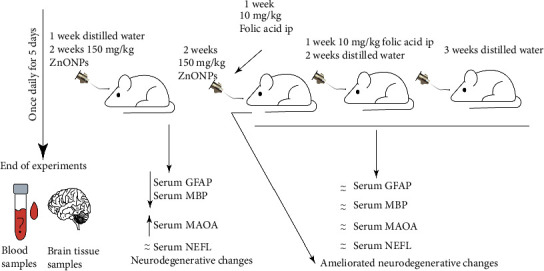
A schematic diagram illustrating the overall procedure and significant discoveries of this research.

Figure 6 illustrates a summary of the various experimental procedures conducted and the main findings of this study.

Tissue from folic + ZnONPs group versus tissue from ZnONPs group revealed significant reduction in all pathology parameters, supporting folic acid's protective role. Control samples provided baseline for distinguishing ZnONP-induced effects from natural variability. Folic acid supplementation alone did not significantly alter granule cell morphology compared to controls, confirming that observed neuroprotection in the folic + ZnONPs group was not due to artifactual changes. The total score of damage in Table 2 indicated that ZnONPs induced minimal damage to the dentate gyrus tissue of the challenged animals.

## 4. Discussion

To address the increasing use and advantages of ZnONPs, it is important to conduct a thorough investigation to elucidate the biological consequences arising from exposure to these nanoparticles. One of these possible consequences is neurotoxicity, which is the negative impact on the nervous system due to exposure to toxic substances [20]. This can manifest in a variety of ways, including behavioral impairments and alteration in the levels of neuronal biomarkers. As a result, this study has investigated neurotoxicity due to oral exposure to ZnONPs in rats and potential protective effect of folic acid. The toxic effects were evaluated by measuring serum levels of four neuronal biomarkers GFAP, MAOA, MBP, and NEFL.

GFAP is considered as an indicator of astroglial activation and astrocytosis [21]. Astrocytes, which are a type of glial cell, have several important functions in the brain. These include modulating synapses, supporting neurons, maintaining homeostasis, and repairing tissue [21, 22]. Intoxicated animals expressed significantly lower levels of GFAP in serum compared to animals that had folic acid as a prophylaxis and to control with or without folic acid. According to research, the levels of GFAP may fluctuate during neurotoxicity that depends on factors such as the type, severity, and duration of the toxic exposure [21]. For instance, GFAP levels increased in response to acute or chronic exposure to Aβ plaques, which are associated with Alzheimer's disease [21, 22]. Another study reported that exposure to ip ZnONPs for 28 days significantly increased expression of GFAP in brain tissue of male albino rats, while oral curcumin significantly reduced that level [23]. Differences in the findings could have been due to differences in rat species, doses, routes, and durations of exposure. Conversely, GFAP levels may decrease during stressful conditions such as Parkinson's or Alzheimer's disease when harmful astrocytes secrete a factor that induces neurotoxicity [21]. Additionally, GFAP levels may normalize or decrease following the cessation of neurotoxic exposure [24]. It is worth noting that while a decrease in GFAP levels may indicate neurotoxicity in some cases, it is not a definitive or a specific marker. Other factors such as the type of neurotoxin, the stage of the disease, the presence of other biomarkers, and clinical signs should be considered when assessing neurotoxicity.

MAOA is an enzyme that catalyzes the oxidative deamination of amine neurotransmitters, including dopamine and serotonin [25]. It is highly expressed in neural cells and is localized to the outer mitochondrial membrane [25]. Therefore, MAOA is essential for neurotransmitter regulation and for maintaining normal brain function. A previous study that exposed neural PC-12 cells to 22-nm ZnO and 43-nm ZnO reported a significant upregulation of MAOA expression at concentrations greater than or equal to 10 μg/mL ZnONPs [26]. These findings are consistent with the current observation, where the animals treated with nanoparticles without folic acid prophylaxis exhibited significantly lower levels of serum MAOA compared to animals that had prophylaxis and to control. As such, disruption of normal MAOA levels may affect normal brain function and represent neurotoxicity of the selected nanoparticles.

MBP is a critical component of the myelin sheath, which is a protective layer that surrounds and insulates axons. Damage or deficiency to the myelin sheath can result in axonal loss and severe demyelination diseases [27]. In this study, a significant decrease in MBP levels was observed in ZnONPs overdosed rats without prophylaxis, compared to other groups, including overdosed rats with prophylaxis and control animals. These findings suggest that folic acid may have a protective effect against ZnONP-induced neurotoxicity, as it was able to normalize MBP levels to those of the control group. This is consistent with a previous research showing that thymoquinone, an active constituent of Nigella sativa, has a protective effect against acrylamide-induced neurotoxicity, where acrylamide lowered MBP levels and thymoquinone normalized the levels in rats [28]. Similarly, punicalagin, the main polyphenol compound of pomegranate, raised BMP levels in acrylamide intoxicated rats to normal levels [29].

Regarding NEFL in the serum, it can serve as an indicator of neuronal cell death in the brain and can be used as a good biomarker of neurotoxicity because neurofilaments are released when the axon of a neuron is damaged [30]. A study found that rats treated with central nervous system toxicants had increased serum levels of NEFL, indicating neuronal cell death in the brain [31]. Similarly, another study reported that NEFL levels in the cerebrospinal fluid increased during neurotoxicity in pediatric patients, indicating neuronal injury [24]. These findings suggest that NEFL levels could increase during neurotoxicity. Instead, the presented outcomes revealed no significant changes in serum NEFL levels in the overdosed rats relative to rats protected with folic acid and relative to controls. The differences in species, toxic agent, doses, and durations of exposure could have contributed to the current insignificant changes in this neuromarker level.

Histopathological findings revealed that ZnONPs induced neuronal damage in the dentate gyrus part of hippocampus, including shrinkage, vacuolation, and cell death. These findings are consistent with those of a previous study, which reported that ZnONPs induced incomplete neural tube closure through various cell death modes, including neuronal apoptosis and autophagy [32]. Karyolysis and ghost cells suggest irreversible cell death, which resulted from the ability of ZnONPs to trigger caspase-3 activation, resulting in DNA fragmentation and necrosis [33]. Overall, the granule cells exhibited greater acidophilic staining than those in all other groups, suggesting a possible loss of nucleic acids and increased cytoplasmic protein content, indicative of cell injury. Interestingly, folic acid administration partially ameliorated toxicity, preserving some neuronal structure with a few ghost cells. Folic acid possesses neuroprotective functions by preventing neural cell apoptosis. It protected neuronal cells from aluminum-maltolate-induced apoptosis by preventing the downregulation of miR-19 downstream PTEN/AKT/p53 pathway [34]. Findings from rats that had folic acid alone and from control rats exhibited normal hippocampal morphology, confirming baseline tissue health within the current study setting.

## 5. Conclusions

This study examined the neurotoxicity resulting from excessive oral exposure to ZnONPs in rats and evaluated the potential protective effect of folic acid. The research assessed the impact by analyzing the levels of four neuronal biomarkers (GFAP, MAOA, MBP, and NEFL) in rats' serum. The findings indicated that ZnONPs had detrimental effects on all the evaluated biomarkers, except for NEFL. Furthermore, ZnONPs induced neuronal damage in the dentate gyrus area of the hippocampus while folic acid prophylaxis partially ameliorated tissue toxicity, preserving some neuronal structure. Overall, the administration of folic acid as a preventative measure reduced the toxicity of ZnONPs. These findings inspire future research to explore alternative biomarkers of neurotoxicity caused by nanoparticles to enhance our understanding of their mechanism and develop more efficient preventive strategies against their toxicity.

## Figures and Tables

**Table 1:** Scoring criteria for dentate gyrus granule cells.

Feature	Score 0 (normal)	Score 1 (minor)	Score 2 (mild)	Score 3 (moderate)	Score 4 (severe)
Ghost cells	Absent (< 0.1%)	Rare (0.1%–0.9%)	Few (1%–4.9%)	Focal (5%–20%)	Widespread (> 20%)
Pyknosis	Absent	Rare	Few nuclei condensed	Clusters of pyknotic nuclei	Diffuse nuclear shrinkage
Karyolysis	Absent	Rare	Focal pale nuclei	Partial nuclear dissolution	Complete nuclear fading

**Table 2:** Semiquantitative histopathology scores across experimental groups.

Group	Ghost cells	Pyknosis	Karyolysis	Total score
ZnONPs	0.15 ± 0.02^∗^	0.40 ± 0.04^∗^	0.44 ± 0.15^∗^	0.33 ± 0.07^∗^
Folic + ZnONPs	0.11 ± 0.02^∗#^	0.12 ± 0.06^#^	0.09 ± 0.04^#^	0.11 ± 0.04^#^
Folic	0.04 ± 0.02	0.13 ± 0.07	0.14 ± 0.04	0.10 ± 0.04
Control	0.03 ± 0.01	0.14 ± 0.02	0.12 ± 0.06	0.10 ± 0.03

*Note:* Interpretation: 0–0.9: normal/minimal damage; 1–1.9: mild pathology; 2–2.9: moderate damage; 3: severe degeneration.

^∗^vs control (*p* < 0.05).

^#^vs ZnONPs (*p* < 0.05).

## Data Availability

The data that support the findings of this study are available from the corresponding author upon reasonable request.
